# Supplementary material for: Association between oral microbiome and five types of respiratory infections: a two-sample Mendelian randomization study in east Asian population
Source: Front Microbiol. 2024 Apr 10;15:1392473. doi: 10.3389/fmicb.2024.1392473 (PMC11039966; doi:10.3389/fmicb.2024.1392473)
Supplement: Supplementary file 1 [file Data_Sheet_1.zip › Supplementary Materials/MR plots of saliva/Pneumonia/s__Capnocytophaga_leadbetteri_mgs_2973/forest.pdf]

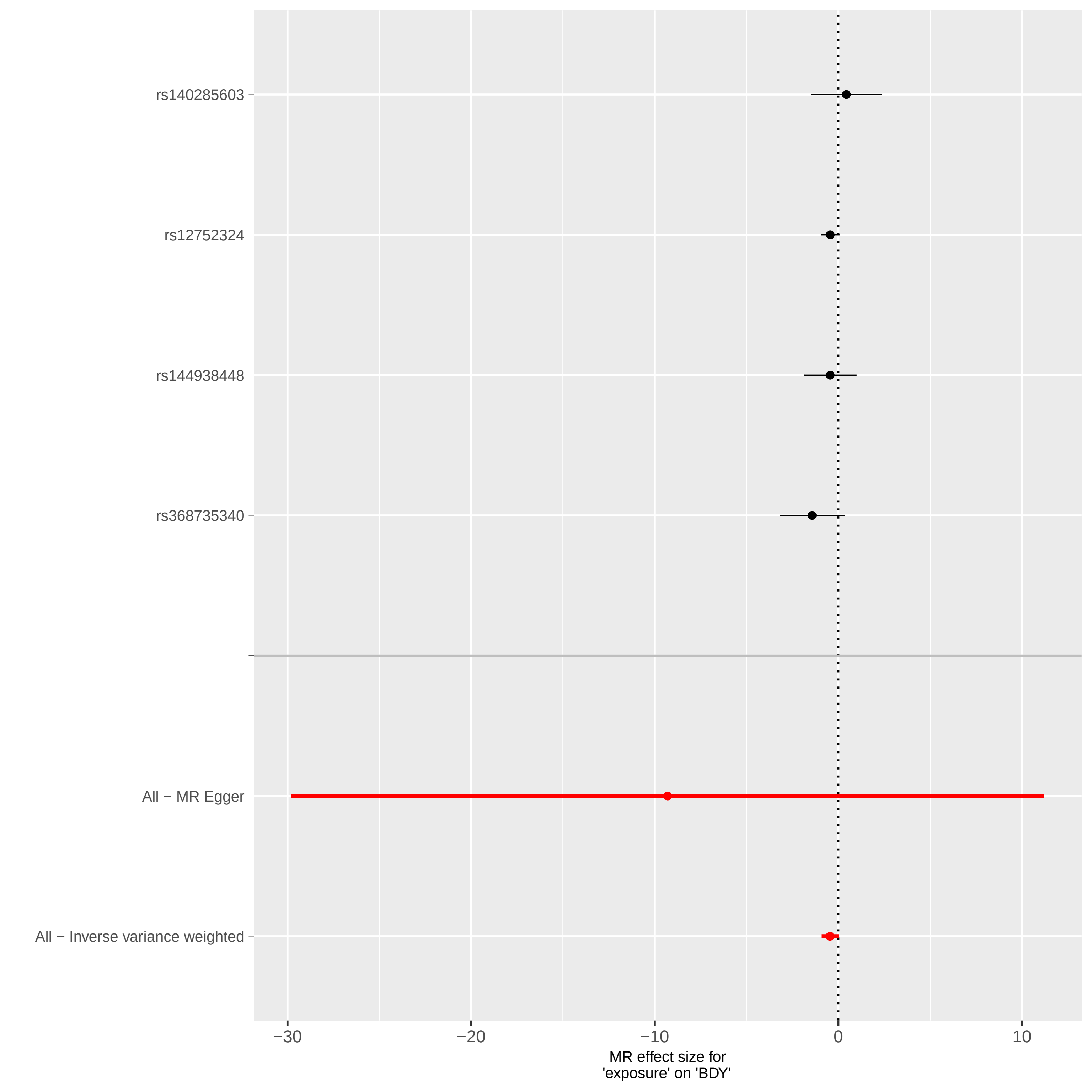
rs140285603
rs12752324
rs144938448
rs368735340
All − MR Egger
All − Inverse variance weighted
−30
−20
−10
0
10
MR effect size for
'exposure' on 'BDY'
